# Mental Health of Parents of Special Needs Children in China during the COVID-19 Pandemic

**DOI:** 10.3390/ijerph17249519

**Published:** 2020-12-18

**Authors:** Sui-Qing Chen, Shu-Dan Chen, Xing-Kai Li, Jie Ren

**Affiliations:** School of Education, Guangzhou University, Guangzhou 510006, China; chensq@gzhu.edu.cn (S.-Q.C.); 2111908009@e.gzhu.edu.cn (S.-D.C.); 2111908089@e.gzhu.edu.cn (X.-K.L.)

**Keywords:** mental health, COVID-19, parents of different types of special needs children, parenting stress, perceived social support

## Abstract

We assessed the mental health of parents (N = 1450, *M*_age_ = 40.76) of special needs children during the COVID-19 pandemic. We conducted an online survey comprising items on demographic data; two self-designed questionnaires (children’s behavioral problems/psychological demand of parents during COVID-19); and four standardized questionnaires, including the General Health Questionnaire, Perceived Social Support, Parenting Stress Index, and Neuroticism Extraversion Openness Five Factor Inventory. The results showed that there were significant differences among parents of children with different challenges. Parents of children with autism spectrum disorder were more likely to have mental health problems compared to parents whose children had an intellectual disability or a visual or hearing impairment. Behavioral problems of children and psychological demands of parents were common factors predicting the mental health of all parents. Parent–child dysfunctional interactions and parenting distress were associated with parents of children with autism spectrum disorder. Family support, having a difficult child, and parenting distress were associated with having children with an intellectual disability. It is necessary to pay attention to the parents’ mental health, provide more social and family support, and reduce parenting pressures.

## 1. Introduction

Since the end of 2019, the severe acute respiratory illness caused by the novel coronavirus (COVID-19) has swept across the world. The World Health Organization declared the coronavirus pandemic as a public health emergency of international concern. Faced with such a critical situation, the Chinese government issued nationwide emergency policies in a short time, including shutting down schools and working places, home quarantine, and other public requirements to limit population mobility. Although these policies could effectively cut off the spread of the virus, these measures are associated with mental health problems, such as anxiety, self-reported stress, and disturbed sleep [1,2]. Many studies have reported the mental health status of medical staff and the general public; however, few have focused on more vulnerable groups such as children with disabilities (i.e., special needs children) and their parents.

In China, there are millions of special needs children, but both them and their parents are still on the edge of social concern. As noted by studies, having a special needs child refers to a long-term challenges for parents, no matter what type and degree of disability the special needs children have [3]. During the COVID-19 pandemic, the nationwide home quarantine that caused school and rehabilitation training institution closure required millions of children with disabilities to stay at home for months [4]. Some children with disabilities might experience behavioral regression and extensive problems because of their inability to access daily school education, rehabilitation training, personalized intervention, and treatments. Instead, parents have to undertake multiple tasks, including parenting, education, rehabilitation, and training, which could increase their parenting stress and cause mental health problems.

According to previous studies, caring for special needs children causes many problems among parents on ordinary days; however, parents with special needs children experience more physical, social, and emotional challenges compared to parents of typically developing children [5]. They also face a greater risk of psychological burden and experience various mental health problems such as anxiety [4,6], depression [3], marital discord [7], and sleep problems [8]. Parents with differently challenged special needs children may display diverse types of mental health problems; however, anxiety and depression are the most common [6,9]. The anxiety and depression scores of parents of special needs children were significantly higher than those of typically developing children [9], whereas the rating of anxiety and depression were always moderately in agreement across parents and children [10,11]. Studies also suggested that parents of children with autism spectrum disorder almost always experience more mental health problems than the other forms of handicaps, such as intellectual disabilities [10,12].

Prior studies have addressed some predicted factors among the mental health of parents of special needs children, such as economic and social status [13], unemployment [14], parenting stress [15,16], children’s behavior problems [17,18] and social support [19,20]. In general, parents of special needs children have to continually seek for more special treatment, medical equipment and other educational services, which more likely to lead to higher economic burden [21]. In the meantime, one of the parents should always be a main caregiver for the special needs child which may cause an overall decline in the family’s ability to work and reduce family income [22]. Therefore, parents of special needs children were always experience poverty because of lower economic and social status or unemployment as compared to their counterparts [9], which exacerbates mental health problems [22]. In addition, the serious behavioral and emotional problems of special needs children, and the pressure to raise such children were main challenge for parents [17,18,23]. Compared to typically developing children, special needs children are more likely to exhibit externalized behaviors, such as difficulties communicating, poor social relationships, and oppositional defiant disorder [24]. The range of serious behavioral problems continually strengthen the parental stress and trigger parents into a poor mental health cycle. Even the frequent regulatory problems of children, such as sleep problems, are also associated with worse mental health condition among parents [25]. It is noted that children’s behavior problems and parenting stress always occur together to predict the mental health of parents. Furthermore, other studies have reported that social support was an important factor for reducing parenting stress and promoting their ability to cope with mental health problems [19,20]. Unfortunately, parents of special needs children are often excluded from society and public concern, even though positive attitudes of raising special needs children among parents is always related to more social support [20]. These risk factors strengthen mental health problems among parents of special needs children.

Based on the above research, we speculated that the mental health problems of parents of children with disabilities may be magnified during the COVID-19 pandemic. These parents may require mental health interventions through appropriate measures; therefore, it is necessary to comprehensively estimate these parents’ mental health status and the key predicting factors. According to reports conducted by the Chinese official institute [26], there are three main types of disabilities among children in China: autism spectrum disorder, intellectual disabilities, and visual or hearing impairments. This study focuses on the parents of three main types of disabilities among children.

## 2. Methods

### 2.1. Participants and Procedure

#### 2.1.1. Procedure

Owing to the home quarantine policy, the questionnaire survey was published on the internet. The online survey targeted the parents of special needs children in Guangdong Province in China and was conducted from 18 February to 22 February 2020. Participants were sampled by stratified random sampling method, recruited through special education schools and special education agencies in Shenzhen, Guangzhou, Shaoguan, Dongguan, Foshan, Huizhou, and 21 other cities or regions. Parents could see this survey and answer the questionnaire by scanning the two-dimensional barcodes on their cellphones or clicking the relevant link of the questionnaire address on their computers. The questionnaire was anonymous to protect the participants, and it was completely voluntary and noncommercial. All the procedures involving human participants were reviewed and approved by the research ethics committee in the School of Education at Guangzhou University (Protocol Number: GZHU2020004).

#### 2.1.2. Participants

A total of 1898 responses were received, of which 1450 were valid (parents of children with autism, intellectual disability, or visual impairment and hearing impairment), with a valid rate of 76.40%. The average age of parents was 40.76 years (SD = 5.84), and there were 401 (27.66%) male participants (*M*_age_ = 43.23, SD = 5.98) and 1049 (72.34%) female participants (*M*_age_ = 39.82, SD = 5.50). According to the classification of special needs children by the China Disabled Persons’ Federation, a total of 454 (31.30%) parents’ children had autism spectrum disorder, with the average age of children being 11.38 years (SD = 3.34). There were 703 (48.50%) parents who had children with an intellectual disability, with the average child’s age of 12.51 years (SD = 3.17). A total of 293 (20.20%) parents had children with a visual or hearing impairment, with the average age of children being 12.47 years (SD = 3.45).

### 2.2. Measures

#### 2.2.1. Demographic Information

Demographic information included sex, age, family monthly income, working state during COVID-19, and so on. Children’s information about gender, age and type of disability were also collected.

#### 2.2.2. Behavioral Problems of Children during COVID-19

We employed a self-designed questionnaire to measure the behavioral problems of children with disabilities during the COVID-19 pandemic. The questionnaire included 6 questions, and each question was scored based on a 4-point Likert scale, ranging from 1 (no) to 4 (many). The exploratory factor analysis showed that the total KMO (Kaiser-Meyer-Olkin) value was 0.75, and the Bartlett’s test of sphericity (*χ^2^* = 2256.69, *df* = 15, *p* < 0.001), which indicated that principal component analysis of the data with Varimax rotation could proceed. Two factors were obtained: Factor 1 is named a regulatory problem (including unwillingness to wear mask, unwillingness to wash hands), of which the Cronbach’s alpha was 0.68. Factor 2 is named as an externalized problem (including asking to go out, sleep problems, eating problems, and mood swings), of which the Cronbach’s alpha was 0.74. Both factors accounted for 63.55% of the questionnaires. The Cronbach’s alpha of the whole scale was 0.76, indicating satisfactory internal consistency in all items. Overall, the confirmatory factor analysis of the questionnaire showed that the model fit well (*χ^2^*/*df* = 4.25, CFI (Comparative Fit Index) = 0.99, TLI (Tucker-Lewis Index) = 0.98, SRMR (Standard Root Mean-Square Residual) = 0.02, RMSEA (Root-Mean-Square Error of Approximation) = 0.05), indicating that the questionnaire was valid.

#### 2.2.3. Psychological Demand of Parents during COVID-19

We employed a self-designed questionnaire to measure the psychological demand of parents of children with disabilities. The questionnaire included 10 questions. Each question was scored based on a 4-point Likert scale, ranging from 1 (inconsistent) to 4 (consistent). The exploratory factor analysis showed that the total Kaiser-Meyer-Olkin value was 0.83, and the Bartlett’s test of sphericity (*χ*^2^ = 4516.19, *df* = 45, *p* < 0.001), which indicated that principal component analysis of the data with Varimax rotation could proceed. Two factors were obtained: Factor 1 was named as an emergency demand for COVID-19 pandemic, such as “I would hoard more supplies (like masks)”, “I would frequently confirm information about the pandemic”, of which the Cronbach’s alpha was 0.78. Factor 2 was named as demands for taking care of the child, such as “I have to deal with child’s behavioral problems”, “I need others to provide special equipment for my child with disabilities”, of which the Cronbach’s alpha was 0.78. These factors accounted for 56.52% of the questionnaires. The Cronbach’s alpha of the whole scale was 0.79, indicating satisfactory internal consistency in all items. Overall, the confirmatory factor analysis of the questionnaire showed that the model fit well (*χ*^2^/df = 3.89, Comparative fit Index = 0.98, Tucker-Lewis index = 0.97, Standard root mean-square residual = 0.02, Root -mean-square error of approximation = 0.04), indicating that the questionnaire was valid.

#### 2.2.4. General Health Questionnaire-12 (GHQ-12)

The GHQ-12 is a mental health screening self-report that identifies whether participants have mental health problems and estimates the magnitude. It is valid, reliable, and utilized around the world [27]. The questionnaire included 12 items (6 positive and 6 negative questions), such as “I am able to concentrate when doing things” (positive), “I am in a happy mood” (positive), “I always feel stressed” (negative), and “I lose confidence” (negative). Each item was scored on a 4-point Likert scale, ranging from 1 (less than usual) to 4 (much more than usual). We used a “0-0-1-1” scoring method (bimodal scoring method) to add all the scores [28,29]. Under GHQ scoring standard, the total score ≥3 indicated poor mental health of parents with special needs children [28]. In this study, the Cronbach’s alpha of this scale was 0.90.

#### 2.2.5. Perceived Social Support (PSS)

The PSS is a questionnaire that assesses the magnitude of support. It consists of three subscales: Family Support, Friend Support, and Other Necessary Support [30]. Each item was scored based on a 7-point Likert scale, ranging from 1 (strongly disagree) to 7 (strongly agree). The Family Support Subscale included four items, such as “My family can actually help me”, “I can talk to my family about my problems”, etc. In this subscale, the Cronbach’s alpha was 0.92. The Friend Support Subscale included four items, such as “My friends can actually help me”, “I can talk to my friends about my problems”, etc. In this subscale, the Cronbach’s alpha was 0.92. The Other Necessary Support subscale included four items, such as “Some people (leaders, relatives, colleagues, etc.) will show up by my side when I have problems”, “I can share happiness and sadness with some people (leaders, relatives, colleagues, etc.)”, etc. In this subscale, the Cronbach’s alpha was 0.87. A total score was created by summing the three subscale scores, with higher scores indicating more perceived support. In this study, the Cronbach’s alpha of the PSS was 0.95.

#### 2.2.6. Parenting Stress Index-Short Form 15 (PSI-SF-15)

The PSI-SF-15 is a questionnaire that measures parenting stress and burden. It consists of three subscales: parenting distress, parent–child dysfunctional interaction, and difficult child [31]. Each item was scored based on a 5-point Likert scale, ranging from 1 (strongly disagree) to 5 (strongly agree). The Parenting Distress Subscale included five items, such as “I cannot deal with new things”, “I feel like I can’t always do what I want to do”, etc. In this subscale, the Cronbach’s alpha was 0.85. The parent–child dysfunctional interaction subscale included five items, such as “My children seldom do anything that makes me feel more satisfied”, “I don’t think my children like me at most of time”, etc. In this subscale, the Cronbach’s alpha was 0.85. The Difficult Child Subscale included five items, such as “What my children do sometimes makes me angry”, “I feel that my child is very emotional and easy to worry”, etc. In this subscale, the Cronbach’s alpha was 0.90. A total score was created by summing the three subscale scores to indicate overall parenting stress. In this study, the Cronbach’s alpha of the PSI-SF-15 was 0.93.

#### 2.2.7. Neuroticism Extraversion Openness Five Factor Inventory (NEO-FFI)

The NEO-FFI is a questionnaire that measures personality traits and comprises five subscales: Neuroticism, Extraversion, Openness, Agreeableness, and Conscientiousness [32]. Each subscale includes 12 items, and every item was scored based on a 5-point Likert scale, ranging from 1 (strong disagreement) to 5 (strong agreement). Neuroticism examples include, “I’m not a worried person”, “I often feel inferior to others”, etc. Neuroticism is a variable highly related to mental health. Controlling neuroticism can better understand the mental health of parents during the pandemic. The total score of Neuroticism Subscales was created by summing 12 items. In this study, the Cronbach’s alpha of the NEO-FFI was 0.73.

### 2.3. Data Analysis

SPSS version 26.0 (IBM, Armonk, NY, USA) and Mplus 8.3 were utilized to analyze the data. Firstly, Mplus 8.3 was used for confirmatory factor analysis to determine whether the structural validity of two self-designed questionnaires was reliable. Then, descriptive analyses were conducted to illustrate participants’ demographic information. A one-way analysis of variance was conducted to compare the mental health scores among the parents with three types of special needs children. Finally, Pearson’s correlation coefficients and hierarchical linear regression analyses were performed to analyze the factors predicting the mental health of different types of parents. The rationale for the order of the variables was the theoretical framework stated in the Introduction: economic and social status, unemployment, and other demographic variables would predict parents’ mental health, followed by parenting stress and social support.

The parents with three types of special needs children were analyzed separately, and three hierarchical two-model multiple linear regression models were developed. Block One included demographic variables, such as sex, age, family monthly income, and working state during the COVID-19 pandemic. Neuroticism subscales were also used as a control variable in Block One. Block Two included behavioral problems of the children, psychological demand on the parents, parenting stress (including parenting distress, parent–child dysfunctional interactions, difficult child), and perceived social support (including family support, friend support, the other necessary support). To minimize the possible effects of multicollinearity, the independent variables and moderators were centered at their mean. A two-tailed test with *p* < 0.05 was considered significant.

## 3. Results

### 3.1. Parents Demographics and General Mental Health Status

Table 1 summarizes parents’ demographics. According to the GHQ-12 classic scoring criteria, a score over 3 indicated that parents’ mental health was poor. In this study, 1058 (73%) parents had good mental health and the remaining 392 (27%) had poor mental health.

A one-way analysis of variance was conducted to compare the mental health scores among the three types of parents with special needs children. A Kolmogorov–Smirnov test showed that the distribution of GHQ did conform to the normal distribution (*p* < 0.001). Combined with the P–P plots, the data distribution can be considered as a slightly positively skewed distribution [33]. As previous studies have reported, one-way analysis of variance can be used when the sample size is greater than 50 [34]. In this study, a total of 1450 individuals participated, therefore the one-way analysis of variance could be utilized. Levene’s test of homogeneity of variance showed that the variance of each group was not homogeneous (*p* < 0.001); Tamhane’s T2 was used to perform multiple comparisons.

Overall, the results showed that there were significant differences in the mental health of parents with different types of special needs children (*F* (2, 1447) = 4.05, *p =* 0.02, *η^2^* = 0.01). Parents of children with autism spectrum disorder were more likely to have mental health problems compared to parents of children with a visual or hearing impairment (*p* = 0.03). There were also marginal significant differences between parents of children with autism spectrum disorder and intellectual disabilities (*p* = 0.08). However, there was no significant difference between parents of children with an intellectual disability and those with a visual or hearing impairment (*p* = 0.66). Ranked by mean mental health scores, parents of children with autism spectrum disorder (*M* = 2.88, *SD* = 3.43) was first, followed by an intellectual disability (*M* = 2.45, *SD* = 3.25) and a visual or hearing impairment (*M* = 2.24, *SD* = 2.99). 

### 3.2. Mental Health Status Among Parents of Children with Autism Spectrum Disorder

Pearson’s correlation coefficient analyses were performed to explore the factors related to parents’ mental health. The results showed that parents’ mental health was positively related to behavioral problems in children (*r* = 0.22, *p* < 0.001), psychological demand of parents (*r* = 0.31, *p* < 0.001), parenting distress (*r* = 0.38, *p* < 0.001), parent–child dysfunctional interaction (*r* = 0.36, *p* < 0.001), and difficult child (*r* = 0.27, *p* < 0.001); whereas it was negatively related to family support (*r* = −0.23, *p* < 0.001), friend support (*r* = −0.18, *p* < 0.001), and other necessary support (*r* = 0.22, *p* < 0.001).

Hierarchical linear regression analyses were used to explore the factors predicting parents’ mental health, and parents’ mental health scores were the dependent variable (see Table 2). Demographic variables were entered in the first model as control variables, and the second model included behavioral problems of children, psychological demands of parents, parenting stress (including parenting distress, parent–child dysfunctional interactions, difficult child), and social support (including family support, friend support, and other necessary support). The maximum variance inflation (VIF) was 4.57, indicating that there was no multicollinearity between independent variables.

In the first model, family monthly income, working state during the COVID-19 pandemic, children’s sex, and neuroticism were the significant predictors of mental health, which accounted for 10.5% of the variance. In the second model, family monthly income, neuroticism, behavioral problems of children, psychological demand of parents, parenting distress, and parent–child dysfunctional interaction showed a significant relationship with mental health, with a total of 24.00% of the variance explained.

### 3.3. Mental Health Status Among Parents of Children with an Intellectual Disability

Pearson’s correlation coefficient analyses were performed to explore the factors related to parents’ mental health. The results showed that the mental health of parents of children with an intellectual disability was positively related to behavioral problems in children (*r* = 0.22, *p* < 0.001), psychological demand of parents (*r* = 0.26, *p* < 0.001), parenting distress (*r* = 0.28, *p* < 0.001), parent–child dysfunctional interaction (*r* = 0.27, *p* < 0.001), and difficult child (*r* = 0.26, *p* < 0.001); whereas it was negatively related to family support (*r* = −0.28, *p* < 0.001), friend support (*r* = −0.17, *p* < 0.001), and other necessary support (*r* = −0.15, *p* < 0.001).

Hierarchical linear regression analyses were conducted to explore the factors predicting parents’ mental health, and parents’ mental health scores were the dependent variable (see Table 3). Demographic variables were entered in the first model as control variables, and the second model included behavioral problems of children, psychological demands of parents, parenting stress (including parenting distress, parent–child dysfunctional interactions, difficult child), and social support (including family support, friend support, and the other necessary support). The maximum variance inflation (VIF) was 3.05, indicating that there was no multicollinearity between independent variables.

In the first model, children’ age and neuroticism were the significant predictors of mental health, which accounted for 3.40% of the variance. In the second model, children’ age, neuroticism, behavioral problems of children, psychological demand of parents, family support, parenting distress, and difficult children showed a significant relationship with mental health, with a total of 18.60% of the variance explained.

### 3.4. Mental Health Status Among Parents of Children with a Visual or Hearing Impairment

Pearson’s correlation coefficient analyses were performed to explore the factors related to parents’ mental health. The results showed that the mental health of parents of children with a visual or hearing impairment was positively related to behavioral problems in children (*r* = 0.19, *p* < 0.001), psychological demand of parents (*r* = 0.32, *p* < 0.001), parenting distress (*r* = 0.32, *p* < 0.001), parent–child dysfunctional interaction (*r* = 0.28, *p* < 0.001), and difficult child (*r* = 0.32, *p* < 0.001); whereas it was negatively related to family support (*r* = −0.16, *p* < 0.001), friend support (*r* = −0.17, *p* < 0.001), and other necessary support (*r* = −0.14, *p* < 0.001).

Hierarchical linear regression analyses were conducted to explore the factors predicting parents’ mental health, and parents’ mental health scores were the dependent variable (See Table 4). Demographic variables were entered in the first model as control variables, and the second model included the behavioral problems of children, psychological demands on parents, parenting stress, (including parenting distress, parent–child dysfunctional interactions, difficult child), and social support (including family support, friend support, and other necessary support). The maximum variance inflation (VIF) was 2.63, indicating that there was no multicollinearity between independent variables.

In the first model, sex and neuroticism were significant predictors of mental health, which accounted for 7.20% of the variance. In the second model, sex, neuroticism, behavioral problems of children, and psychological demand of parents showed a significant relationship with mental health, with a total of 21.3% of the variance explained.

## 4. Discussion

This study found that 27% of parents with special needs children experienced mental health problems, which differed depending on the different types of disability in the children. Parents of children with autism spectrum disorder were more likely to have mental health problems compared to parents of children with an intellectual disability and a visual or hearing impairment, which is consistent with previous research [35,36].

### 4.1. Similarities Between the Three Groups of Parents of Special Needs Children

Firstly, neuroticism was always significant in the three hierarchical linear regression models. As a control variable, neuroticism is a stable factor that impacts on mental health. To some extent, it could be used to control the impact of participants’ emotional state before the COVID-19 pandemic. The significant results concerning neuroticism confirmed that independent variables reported in this study could indeed be used to predict mental health among parents of special needs children during the COVID-19 pandemic.

Secondly, behavioral problems of children were the main factors predicting mental health among parents. During the COVID-19 pandemic, the nationwide home quarantine prevented special needs children from not only accessing normal school education, but also rehabilitation training, personalized intervention, and treatments [37]. This may lead to behavioral regression and extensive problems in special needs children. In this study, the three groups of special needs children had regulatory problems (such as an unwillingness to wear masks and unwillingness to wash hands) and externalized problems (such as asking to go out and mood swings). Combined with previous studies, special needs children’s behavioral problems are always closely associated with parenting stress, and jointly predict the mental health among parents of special needs children. 

Finally, the psychological demand of parents also predicts mental health among all parents. On the one hand, most parents were similar to the general public, in that they would frequently confirm information about the pandemic and hoard more supplies (such as masks) [38]. Some studies have shown that such behaviors are normal, although it could also lead to the emergence of a poorer general mental health condition of parents of special needs children [1]. On the other hand, parents of special needs children need to continually seek out special treatment, medical equipment, and other services about how to deal with child’s behavior, which would increase their psychological burden. The pandemic would prevent parents from seeking solutions to deal with the behavioral problems among special needs children. 

### 4.2. The Differences Between the Three Groups of Parents of Special Needs Children

Previous studies have shown that the behavioral problems of special needs children are determined by the type and degree of the child’s disorder. Among the behavioral problems of autism disorders, the core issues include communication impairments and interference with restrictive behaviors [17]. In this study, we reported six behavioral problems in children, such as unwillingness to wear masks, unwillingness to wash hands, asking to go out, sleep problems, eating problems, and mood swings. These may be important manifestations of restrictive behaviors in children with autism during the COVID-19 pandemic. In normal times, children with autism may also experience tantrums, impatience, and anxiety because they cannot go outside or are not used to obeying the new rules (such as wearing a mask). However, at the same time, children with autism spectrum disorder are not good at communicating with parents and solving problems independently [39]. Consequently, parents have to exert more time and resources, which may increase more parenting stress. From this perspective, children’s behavioral problems are closely related to parenting pressure, and jointly predict mental health among parents. Furthermore, behavioral problems in children with autism are also associated with parent–child dysfunctional interactions among parents. Studies have shown that there is a two-way connection between parent–child relationships and behavioral problems in children [40]. This means that children’s behavioral problems could predict the tendency or change in the emotional quality of parent–child relationships. In other words, parents have to work harder during these parent–child interactions [41]. When repeated and restrictive behavioral problems are difficult to solve, parents tend to be more directive and control by employing verbal or corporal punishments, which decrease closeness and increase parent–child dysfunction [42]. Over time, this not only strengthens behavioral problems in children, but also results in increased mental health problems of parents.

Among parents of children with an intellectual disability, behavioral problems were not only related to parenting distress, but also to difficult children. Compared with the restrictive behaviors of children with autism, children with an intellectual disability are mainly impaired in intellectual functioning and have poor performance in evidencing of impaired adaptive functioning, such as conceptual, social communication, and practical self-care domains [43]. Such children need more sustained, long-term, and repeated training, which could gradually improve their daily behavior. However, during the COVID-19 pandemic, policies not only interrupted children from receiving rehabilitation treatment, but also increased their externalized problems in the absence of social activities, increased loneliness, and insufficient information [4]. Furthermore, frequent regulatory problems (such as sleep problems) are also common behavioral problems in children with intellectual disabilities, which usually affect parents’ mental health [44]. Therefore, it is reasonable that the difficulty of parenting and the distress of parenting were used to predict mental health among parents of children with an intellectual disability.

In addition, parents of children with intellectual disabilities were good at seeking family support. It is interesting to note that family support was only significantly associated with mental health among parents of children with an intellectual disability, rather than the other types of parents. In China, the mode of caring for intellectual disabilities children still relies heavily on families [45]. The model is changing, however, and social support systems are gradually being constructed [19,20]. Based on this, the symptoms of special needs children are easily noticed by their parents, who can gather family strength and help children access special treatment and equipment. Family support becomes a protective factor for parents [19,20,46]. However, some studies have showed that parents of children with autism receive less family support, which may result in increased parenting distress, anxiety, depression, and poor mental health among parents [47]. The relationship between social support and mental health in the other types of parents is complex, and requires additional research.

Among parents of children with a visual impairment, we found that neither parenting stress nor social support was significant; however children’s behavioral problems were still significant. Compared to other special needs children, children with a visual or hearing impairment were likely to have fewer behavioral problems. However, during the COVID-19 pandemic, children with a visual or hearing impairment also displayed the six behavioral problems as mentioned above. Moreover, owing to their visual or hearing impairment, these children lag behind typically developing children in speaking, writing, attention, and executive functions [48]. Spoken language and writing skills play important roles in communication; visual attention, executive function, etc., are conducive to planning, problem solving, and the suppression of behavioral problems [49,50]. Therefore, children with a visual or hearing impairment have more behavioral problems, which would increase the psychological demand of parents compared to typically developing children. Overall, the mental health among such parents tended to be consistent with parents of typically developing children.

### 4.3. Implications 

This study has two implications for the parents of special needs children. On the one hand, the results showed that 27% of parents had poor mental health during the pandemic. This suggests that we need to pay more attention to the mental health of parents of special needs children, especially parents of children with autism or intellectual disabilities. On the other hand, for all groups of parents, family support was critical to parents’ mental health; however, the parents of children with autism are receiving insufficient family support. This should be addressed to improve parents’ mental health.

### 4.4. Limitations

There are some limitations that should be considered when interpreting this study’s findings. Firstly, we examined three types of disabilities; thus, the results cannot be generalized to parents of other special needs children. Secondly, all of the outcomes were self-reported, which might lead to recall bias. However, using self-reported scales to measure mental health is common because of its convenience. To enhance the internal validity of the results, future research may use multiple measurement modalities for each variable. Thirdly, because an online questionnaire was used in the study, the computer literacy of respondents might have affected how they responded to the questionnaire. Additionally, we controlled neuroticism to better understand the mental health of parents during the pandemic. To increase the validity of the results, other variables can be added in future studies. Finally, because the data collection took place during the pandemic, without further data collection, we cannot know the long-term impact of discrimination amid the pandemic on the respondents’ psychological distress. Future studies can utilize random sampling strategies with multiple data collection in order to study the impact of discrimination over time.

## 5. Conclusions

In summary, some parents of special needs children had mental health problems during the pandemic. There were significant differences in the mental health of parents depending on the child’s disability. Overall, the behavioral problems of children and the psychological demands of parents were common factors predicting the mental health of all parents. Parent–child dysfunctional interactions and parenting distress were associated with parents of children with autism spectrum disorder. Family support, difficult child, and parenting distress were associated with parents of children with an intellectual disability. It is therefore necessary to provide more social and family support to reduce pressure on parents.

## Figures and Tables

**Table 1:** Demographic information about parents.

Variables	Parents of Children with Autism Spectrum Disorder (*n* = 454)		Parents of Children with Intellectual Disability (*n* = 703)		Parents of Children with Visual or Hearing Impairment (*n* = 293)	
	*n*	%	*n*	%	*n*	%
Sex						
Male	124	27.31	172	24.47	105	35.84
Female	330	72.69	531	75.53	188	64.16
Family monthly income						
Under CNY 5000	220	48.46	400	56.90	207	70.65
CNY 5000–9999	136	29.96	187	26.60	55	18.77
CNY 10,000–14,999	52	11.45	58	8.25	17	5.80
CNY 15,000 and above	46	10.13	58	8.25	14	4.78
Working state						
Normal rework	100	22.03	154	21.91	57	19.45
Home office	85	18.72	104	14.79	43	14.68
No work	269	59.25	445	63.30	193	65.87
Mental health						
Good (GHQ < 3)	315	69.38	518	73.68	225	76.79
Poor (GHQ ≥ 3)	139	30.62	185	26.32	68	23.21

Abbreviations: GHQ: General Health Questionnaire.

**Table 2:** Hierarchical linear regression analysis among parents of children with autism spectrum disorder.

Models	Variables	*B*	*SE*	*β*	*t*	*95*% CI	*R^2^*	*F*
Model 1	Age	0.03	0.04	0.05	0.83	−0.04, 0.1	0.11	80.61 **
	Sex	0.37	0.37	0.05	1.01	−0.35, 1.09		
	Family monthly income	−0.52	0.16	−0.15	−3.29 **	−0.83, −0.21		
	Working state	0.42	0.19	0.01	2.17 *	0.04, 0.80		
	(Child) Age	−0.01	0.06	−0.01	−0.17	−0.12, 0.10		
	(Child) Sex	−0.94	0.44	−0.01	−2.14 *	−1.81, −0.08		
	Neuroticism	1.70	0.31	0.25	5.55 **	1.10, 2.31		
Model 2	Age	0.01	0.03	0.02	0.34	−0.06, 0.08	0.24	10.54 **
	Sex	0.26	0.34	0.03	0.76	−0.41, 0.92		
	Family monthly income	−0.30	0.15	−0.09	−2.02 *	−0.60, −0.01		
	Working state	0.27	0.18	0.07	1.51	−0.08, 0.63		
	(Child) Age	0.05	0.05	0.05	1.04	−0.05, 0.16		
	(Child) Sex	−0.61	0.41	−0.06	−1.48	−1.42, 0.20		
	Neuroticism	0.80	0.31	0.12	2.59 **	0.19, 1.40		
	Behavioral problems (of children)	0.57	0.26	0.10	2.25 *	0.07, 1.07		
	Psychological demand (of parents)	1.07	0.32	0.16	3.31 **	0.43, 1.70		
	Family support	−0.23	0.19	−0.09	−1.17	−0.61, 0.15		
	Friend support	0.17	0.22	0.06	0.76	−0.27, 0.60		
	The other necessary support	−0.32	0.24	−0.12	−1.33	−0.78, 0.15		
	Parenting distress	0.53	0.23	0.14	2.33 *	0.08, 0.98		
	Parent–child dysfunctional interaction	0.68	0.24	0.18	2.84 **	0.21, 1.14		
	Difficult child	−0.24	0.19	−0.08	−1.25	−0.63, 0.14		

Note: all the variables are centered at their mean. * *p* < 0.05; ** *p* < 0.01.

**Table 3:** Hierarchical linear regression analysis among parents of children with an intellectual disability.

Models	Variables	*B*	*SE*	*β*	*t*	*95*%CI	*R^2^*	*F*
Model 1	Age	−0.02	0.02	−0.03	−0.73	−0.06, 0.03	0.03	4.54 **
	Sex	−0.12	0.30	−0.02	−0.40	−0.70, 0.47		
	Family monthly income	−0.11	0.13	−0.03	−0.84	−0.37, 0.15		
	Working state	0.20	0.15	0.05	1.35	−0.09, 0.50		
	(Child) Age	0.08	0.04	0.08	1.99 *	0.00, 0.17		
	(Child) Sex	0.30	0.25	0.04	1.19	−0.19, 0.79		
	Neuroticism	1.10	0.23	0.18	4.81 **	0.65, 1.55		
Model 2	Age	0.01	0.02	0.02	0.56	−0.03, 0.06	0.19	11.71 **
	Sex	−0.06	0.28	−0.01	−0.21	−0.60, 0.48		
	Family monthly income	0.04	0.12	0.01	0.33	−0.20, 0.28		
	Working state	0.10	0.14	0.03	0.70	−0.18, 0.37		
	(Child) Age	0.10	0.04	0.10	2.52 *	0.02, 0.18		
	(Child) Sex	0.20	0.23	0.03	0.87	−0.25, 0.66		
	Neuroticism	0.44	0.23	0.07	1.90 *	−0.02, 0.89		
	Behavioral problems (of children)	0.72	0.27	0.10	2.70 **	0.20, 1.25		
	Psychological demand (of parents)	0.96	0.23	0.15	4.09 **	0.50, 1.42		
	Family support	−0.82	0.15	−0.30	−5.40 **	−1.12, −0.52		
	Friend support	0.07	0.17	0.02	0.39	−0.27, 0.40		
	The other necessary support	0.24	0.17	0.08	1.38	−0.10, 0.58		
	Parenting distress	0.46	0.18	0.12	2.56 *	0.11, 0.81		
	Parent–child dysfunctional interaction	−0.17	0.23	−0.05	−0.76	−0.62, 0.27		
	Difficult child	0.40	0.16	0.13	2.46 *	0.08, 0.71		

Note: all the variables are centered at their mean. * *p* < 0.05; ** *p* < 0.01.

**Table 4:** Hierarchical linear regression analysis among parents of children with a visual or hearing impairment.

Models	Variables	*B*	*SE*	*β*	*t*	95%CI	*R^2^*	*F*
Model 1	Age	−0.02	0.03	−0.05	−0.71	−0.08, 0.04	0.07	4.24 **
	Sex	−0.77	0.37	−0.12	−2.05*	−1.50, −0.03		
	Family monthly income	−0.11	0.21	−0.03	−0.53	−0.53, 0.31		
	Working state	0.25	0.22	0.07	1.14	−0.18, 0.67		
	(Child) Age	0.00	0.05	0.00	0.07	−0.10, 0.11		
	(Child) Sex	−0.18	0.35	−0.03	−0.52	−0.87, 0.50		
	Neuroticism	1.58	0.33	0.27	4.77 **	0.93, 2.23		
Model 2	Age	0.01	0.03	0.03	0.46	−0.04, 0.07	0.21	6.26 **
	Sex	−0.72	0.35	−0.12	−2.05 *	−1.40, −0.03		
	Family monthly income	0.07	0.20	0.02	0.34	−0.33, 0.46		
	Working state	0.18	0.20	0.05	0.88	−0.22, 0.58		
	(Child) Age	−0.02	0.05	−0.02	−0.37	−0.12, 0.08		
	(Child) Sex	−0.21	0.33	−0.04	−0.65	−0.85, 0.43		
	Neuroticism	−0.97	0.37	0.17	2.63 **	0.25, 1.69		
	Behavioral problems (of children)	0.88	0.53	0.09	1.66 *	−0.16, 1.92		
	Psychological demand (of parents)	1.34	0.32	0.24	4.23 *	0.71, 1.96		
	Family support	−0.32	0.21	−0.13	−1.52	−0.72, 0.09		
	Friend support	−0.06	0.20	−0.02	−0.29	−0.46, 0.34		
	The other necessary support	−0.16	0.20	−0.07	−0.80	−0.56, 0.24		
	Parenting distress	0.36	0.26	0.10	1.38	−0.15, 0.87		
	Parent–child dysfunctional interaction	−0.02	0.29	−0.01	−0.08	−0.60, 0.56		
	Difficult child	0.36	0.27	0.11	1.34	−0.17, 0.90		

Note: all the variables are centered at their mean. * *p* < 0.05; ** *p* < 0.01.
